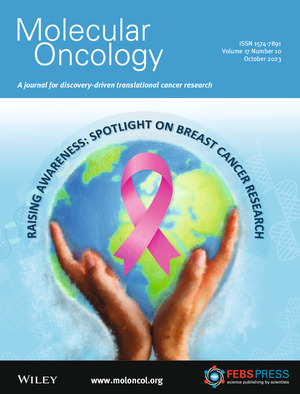# Issue Information

**DOI:** 10.1002/1878-0261.13240

**Published:** 2023-10-05

## Abstract

In light of Breast Cancer Awareness Month, the current issue presents a commentary (pp. 1947–1949) and a method article (pp. 1953–1961) focusing on innovative diagnostic strategies in breast cancer, as well as an array of research articles (pp. 1962–2108) exploring novel breast cancer biomarkers, the clinical impact and molecular intricacies of diverse therapeutic approaches, and challenges related to therapy resistance.

Illustration credit: Sofija Grabuloska.